# ST13-PDGFRβ阳性急性髓系白血病1例报告并文献复习

**DOI:** 10.3760/cma.j.issn.0253-2727.2023.08.011

**Published:** 2023-08

**Authors:** 海荣 仇, 纯 乔, 慧 杨, 睿 郭, 雨 时, 晓丽 赵, 建勇 李, 雨 朱

**Affiliations:** 南京医科大学第一附属医院，江苏省人民医院血液科，南京 210029 Department of Hematology, Jiangsu Province Hospital, the First Affiliated Hospital of Nanjing Medical University, Nanjing 210029, China

2016年WHO造血与淋巴组织肿瘤分型标准将伴嗜酸性粒细胞增多的髓系/淋系肿瘤中具有PDGFRα、PDGFRβ、FGFR1重排或PCM1-JAK2者作为一个单独亚型定义[1]。嗜酸性粒细胞增多是这组疾病中常见且显著的特征，但在某些病例中缺失。受编码特定酪氨酸激酶基因重排的驱动，激酶结构域被激活，导致细胞信号传导失调，从而促进肿瘤细胞增殖和生存。其对酪氨酸激酶抑制剂（TKI）的敏感性已得到广泛认可。PDGFRβ位于染色体5q32上，该基因编码的蛋白质是血小板源性生长因子家族成员的细胞表面酪氨酸激酶受体，属于Ⅲ型酪氨酸激酶受体家族成员，其在胚胎发育、细胞增殖、存活、分化、趋化和迁移中起着重要的调节作用。目前已发现有40多种PDGFRβ重排对手基因。本文报道首例ST13-PDGFRβ融合基因阳性急性髓系白血病（AML）病例并进行文献复习。

## 病例资料

患者，男，34岁，2021年11月无明显诱因出现上腹痛。血常规：WBC 26.82×10^9^/L，中性粒细胞绝对计数15.15×10^9^/L，HGB 121 g/L，PLT 393×10^9^/L。骨髓象：增生明显活跃，原始粒细胞占72.4％，提示为AML部分分化型。免疫分型：原始幼稚细胞占33.0％；表达CD34、CD117、CD13、HLA-DR、CD33、CD71、CD38、CD81、CD11c；部分表达cMPO、CD56、CD123、CD71，提示为伴CD56^+^ AML。染色体核型：46,XY[20]；荧光原位杂交：RUNX1-RUNX1T1融合探针检测阴性；多重PCR筛查56种白血病相关融合基因阴性；髓系相关突变二代测序（NGS）：FLT3-ITD突变阳性（p.S585delinsWMVQVTGSSDNE YFYVDFRA突变比例82.58％）。2021年11月25日起予标准剂量IA方案诱导治疗：伊达比星12 mg/m^2^第1～3天，阿糖胞苷100 mg/m^2^第1～7天（24 h持续静脉滴注）。12月15日评估骨髓形态学：增生活跃，原始细胞占4.0％；流式细胞术检测微小残留病（MRD）：分析2.1％的幼稚细胞群，表达CD34、CD33、CD38、HLA-DR，部分表达CD56，弱表达CD117。Sanger测序法检测FLT3-ITD突变仍为阳性。鉴于患者诱导治疗后缓解质量不高，血细胞恢复困难，于2021年12月29日调整方案巩固治疗：阿扎胞苷75 mg/m^2^ 第1～7天，维奈克拉400 mg第1～28天，吉瑞替尼120 mg第1～28天。2022年2月8日复查骨髓形态学：增生活跃，未见原始幼稚细胞；流式细胞术检测MRD阴性。建议患者行异基因造血干细胞移植，但因客观原因未能进行。后患者继续用同方案巩固治疗2个周期，期间每周期开始前复查骨髓形态学，均为MRD阴性状态。2022年5月自行停止治疗。2022年7月24日于当地医院常规复查发现血常规异常：WBC 60.97×10^9^/L，HGB 91 g/L，PLT 42×10^9^/L；行外周血细胞形态分析：原始细胞41％，幼稚细胞36％，考虑白血病复发，再次收入院。复发后再次筛查白血病相关遗传学背景，56种白血病相关基因多重PCR筛查均为阴性；髓系相关突变NGS检测FLT3-ITD突变阳性（p.S585delinsWMVQVTGSSDNEYFYVDFRA 突变比例10.56％）；染色体核型：46,XY,t（5;22）（q32;q13）[10]（图1A）；进一步FISH检测PDGFRβ重排阳性（1R1G1F：270/300）（图1B）；全转录组mRNA测序检出ST13-PDGFRβ融合基因转录本（图1C），以及DDX41、NF1两种基因突变。ST13-PDGFRβ新融合基因经RT-PCR及Sanger测序得以验证。Sanger测序显示ST13基因的第10外显子和PDGFRβ基因的第11外显子之间存在融合（图1D）。复发后患者已转至他院继续治疗，后因病情恶化死亡。

**图1:**
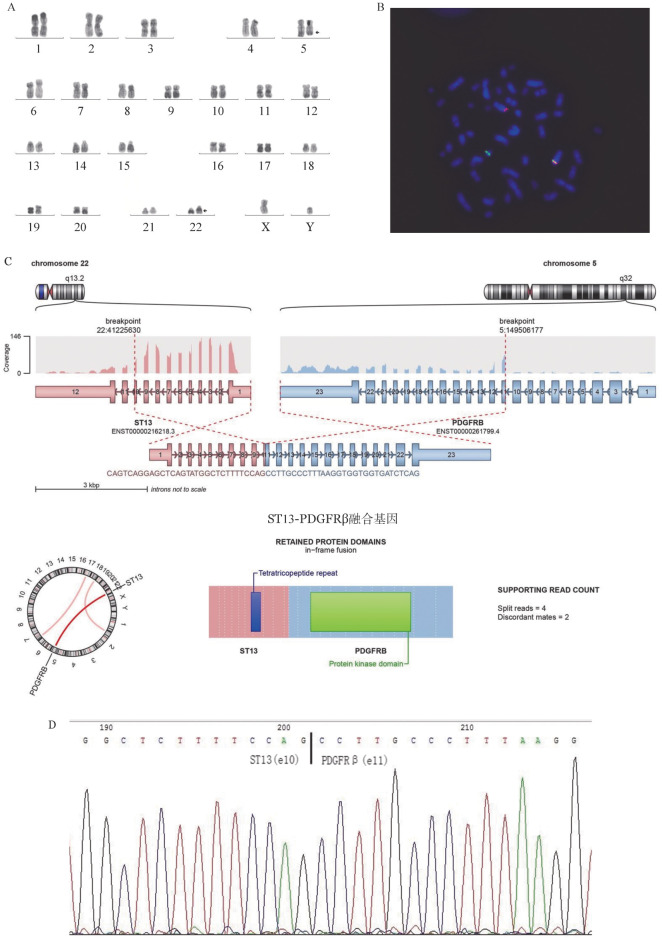
ST13-PDGFRβ融合基因阳性患者细胞遗传学及分子学检测 A 染色体核型检测显示46,XY,t(5;22)(q32;q13)； B 荧光原位杂交检测显示PDGFRβ重排阳性； C 全转录组mRNA测序检出ST13-PDGFRβ融合基因； D Sanger测序证实ST13与PDGFRβ之间存在融合

## 讨论并文献复习

PDGFRβ重排在骨髓增生性肿瘤中的发生率约为1.8％，ETV6是最早被发现的也是最常见的PDGFRβ对手基因[1]–[3]。PDGFRβ重排也可见于急性淋巴细胞白血病，最常见的对手基因是EBF1[4]。本例PDGFRβ重排见于1例FLT3-ITD突变阳性的复发难治AML，患者病程中未见嗜酸性粒细胞增多表现，初诊时为正常核型，8个月复发后核型演变为46,XY,t（5;22）（q32;q13），后经FISH及二代测序及Sanger测序证实为ST13-PDGFRβ融合基因阳性。ST13作为PDGFRβ新的对手基因目前未见各大数据库（Fusion cancer, My Cancer Genome, Atlas of Genetics and Cytogenetics in Oncology and Haematology等）报道。该基因编码的蛋白质是一种衔接蛋白，可介导热休克蛋白HSP70和HSP90的结合。作为HSP70的分子共伴侣，参与HSP70在蛋白质折叠修复等过程中的分子伴侣作用[5]。随着近年来研究深入，发现ST13基因在结肠、胃、乳腺、子宫、卵巢等组织及相应的肿瘤中都有不同程度的表达。

目前已发现40余种PDGFRβ重排的对手基因，我们查阅文献并对44例首次报告病例进行综合分析[6]–[18]。资料显示除1例文献中未提及性别，其余43例中男女比例为36∶7，表现为男性患者显著多于女性，呈男性明显高发的现象，然而由于研究病例数有限，该结论有待后续进一步证实。PDGFRβ重排被报道于多种髓系、淋系肿瘤，其中最常发生在骨髓增殖性肿瘤（MPN）中，发生率约为38.6％（17/44），其次为慢性嗜酸性粒细胞白血病（CEL），发生率约为15.9％（7/44），而检索到的44例患者中仅有4例AML及3例ALL被报道，此外还见于3例慢性粒-单核细胞白血病（CMML）及2例幼年型粒-单核细胞白血病（JMML），总体而言在髓系肿瘤中发生率远高于淋系肿瘤。在有病例资料的患者中88.9％（32/36）的患者WBC>10×10^9^/L，85.4％（35/41）存在嗜酸性粒细胞增多，可见高白细胞和嗜酸性粒细胞增多是此类疾病的常见表现。除6例缺乏治疗信息外，其余病例中89.5％（34/38）接受过TKI治疗，94.1％（32/34）经TKI治疗有效。在接受TKI治疗的患者中，除2例因耐药接受其他治疗外，32例中达到完全分子学缓解（CMR）者9例，达完全遗传学缓解（CCyR）者9例，达完全血液学缓解（CHR）者12例，达部分缓解（PR）者2例；达到CHR及以上疗效者30例（93.8％）。伊马替尼治疗剂量从100～800 mg/d不等，最多见的是以初始剂量400 mg/d 治疗，共有15例，其中有3例因出现血小板或白细胞减低等不良反应而减量，从文献报道可知仅100 mg/d剂量的伊马替尼即可诱导完全缓解。以上数据显示，不论疾病类型如何，PDGFRβ重排阳性患者，TKI均被认为是重要且有效的治疗手段。遗憾的是本例患者复发后尚未使用TKI治疗即因疾病进展死亡。

此外，对手基因所在染色体除13、18、19、21号目前未见报道外其余常染色体均有涉及。其中有4例涉及12p13形成t（5;12）（q32;p13），3例涉及17p11形成t（5;17）（q32;p11.2），虽然在同一断裂位点，但对应不同的对手基因，形成的融合基因也各异。12p13及17p11的高频发生提示此类断裂重排点为敏感位点。在所有44例患者中6例（13.6％）在常规核型分析中未检出相关异常，这是由于PDGFRβ易位常表现为隐匿易位，另有极少数融合基因由缺失或插入易位产生，常规染色体核型分析受肉眼识别限制，敏感性低，容易漏诊。FISH方法应用断裂探针检测基因重排，是此类对手基因众多的基因异常筛选的重要手段，RNA测序亦可作为重要补充，以此提高检出率。尤其对于伴嗜酸性粒细胞增多的髓系/淋系肿瘤，以使更多患者能够受益于TKI治疗。随着研究的深入，文献报道PDGFRβ重排Ph-like ALL患者伴PDGFRβ点突变者表现为对TKI耐药[19]。本例患者具有DDX41、NF1、FLT3三种基因突变。DDX41基因编码蛋白能够同多种剪切体蛋白相互结合，在胚胎发育、细胞生长和分裂中起重要作用。DDX41基因突变是髓系肿瘤伴DDX41胚系易感基因变异亚型诊断重要指标[20]。NF1基因表达产物对RAS信号通路起负调控作用，该基因失活性突变或者拷贝数缺失能够激活RAS信号通路。NF1突变常见于CMML，也可见于AML。FLT3与PDGFRβ一样同属Ⅲ型酪氨酸激酶受体家族成员，在早期造血祖细胞增殖、分化方面起重要作用，FLT3-ITD突变在成人AML中发生率约为30％，具有易复发、预后差等临床特征。目前FLT3靶向治疗已取得实质性进展，可大幅延长患者生存期，新药米哚妥林是一种多靶点TKI，对FLT3、PDGFRα、PDGFRβ等多种酪氨酸激酶均有抑制作用[21]。本例患者同时具有FLT3-ITD及PDGFRβ重排，多靶点抑制剂可能为此类患者的理想选择。
